# The photoreceptive and neuroendocrine pineal organ of Atlantic salmon

**DOI:** 10.3389/fphys.2026.1778109

**Published:** 2026-05-25

**Authors:** Christine Horne, Jon Vidar Helvik, Rita Karlsen, Elsa Denker, Mariann Eilertsen

**Affiliations:** Department of Biological Sciences, University of Bergen, Bergen, Norway

**Keywords:** *aanat2*, choroid plexus, *exorhodopsin*, melatonin, *parapinopsin*, *peropsin*, *retinal G-protein coupled receptor opsin*

## Abstract

Daily and seasonal variations in the periodicity of sunlight are one of the most important cues about the external environment, and nonvisual photoreception is essential for registration and correct timing of many biological processes. Atlantic salmon (*Salmo salar*) is a species where life history transitions are controlled by seasonal changes in light periodicity. In teleost, the pineal organ is photosensitive and produces melatonin, however, the diversity of photoreceptors, their organization and which opsin is responsible for the photic regulation of melatonin synthesis have got little attention in salmon. Based on RNA sequencing and histological profiling of the Atlantic salmon pineal, this study provides an extensive topographic mapping of several nonvisual opsins of the alevin and parr pineal organ in combination with melatonin synthesizing enzymes *arylakylamine N-acetyltransferase* (*aanat2*) and *N-acetylserotonin O-methyltransferase* (*asmt*). The results show that *exorhodopsin* (*exorh*) expressing cells constitute the vast majority of photoreceptors in the pineal organ. These cell types also contain expression of melatonin synthesizing enzymes and are therefore a main regulator of melatonin synthesis in Atlantic salmon, while *parapinopsin b* (*ppb*) most likely has an accessory role in the photic regulation of melatonin synthesis. The majority of the nonvisual photoreceptors are located towards the pineal lumen with their outer segment protruding into this, while *ppb* is located more centrally in the epithelium. *Retinal G-protein coupled receptor* (*rgr*) is expressed in a vast majority of the photoreceptors and is localized together with other opsins like *exorh*, suggesting a photoisomerase function for this opsin. In addition, for the first time, *peropsin* is characterized in the ciliated choroid plexus cells, suggesting the presence of photoreceptive activity in this cerebrospinal fluid producing organ. Overall, expression analysis clearly indicates that the pineal organ is multi-photoreceptive and that melatonin synthesis is linked to photoreceptors expressing *exorh*.

## 1 Introduction

Detection of light, photoreception, is essential for an organism’s ability to orient to the daily and seasonal variations in the external light environment (Bradshaw and Holzapfel, 2007; Foster and Kreitzman, 2014). The non-image-forming nonvisual photoreceptors are responsible for detecting ambient light, and in nonmammalian vertebrates these photoreceptors are located predominantly in the eye, the pineal organ, and the deep brain (Davies et al., 2015, 2010; Perez et al., 2019). Nonvisual photoreceptors entrain the circadian and circannual rhythms and ensure the synchronization of physiology, behavioral patterns, and gene expression to the environment (Perez et al., 2019). Daily variations of light impact the organism on many levels, including regulation of the endocrine system. Melatonin, the time-keeping hormone, is synthesized in the teleost pineal organ in direct response to external light conditions (Axelrod, 1974; Falcón, 1999), but the organization of photoreceptor cell types and which cells are synthesizing melatonin is not fully understood. Understanding the photoreceptive regulation of melatonin production in the pineal organ is of special interest in the Atlantic salmon, where seasonal rhythmicity of light–dark periods drives development and life history transitions.

Light enters directly into the brain of salmonids through a transparent area above the pineal complex, referred to as the “pineal window”, where the skull is thinner and the skin has less pigmentation (Omura and Oguri, 1969). The optical properties of the pineal window have been studied in Atlantic salmon, and light transmission has been measured to be 3%, between 500 and 700 nm in Atlantic salmon (Nordtug and Berg, 1990). In addition, light stimuli have been shown to activate the neuronal immediate *c-fos* expression in regions with nonvisual photoreceptors in the Atlantic salmon deep brain (Eilertsen et al., 2021). The pineal complex consists of the pineal organ and the associated parapineal organ which is much smaller and located close to the diencephalic habenula. The pineal organ is an elongated structure lying on top of the telencephalon just beneath the skull. It evaginates from the diencephalic roof and is attached to the diencephalon by the pineal stalk (Ekström and Meissl, 2003; Falcón, 1999). The pineal epithelium consists of pineal photoreceptors (rod-like and cone-like), neurons, Muller-like glia cells, microglia, macrophages, RPE-like cells, and supportive cells (Falcón and Muñoz-Cueto, 2024; Shainer et al., 2019; Zheng et al., 2024). In many species, the epithelium is strongly folded, almost covering the central lumen of the pineal organ (Ekström and Meissl, 1997; Omura and Oguri, 1969). Pineal photoreceptors transmit light information through two signaling modes, the neural mode and the neuroendocrine mode. As in the retina, neural mode photoreceptors are characterized by having synaptic connections with second-order neurons and are believed to transmit information through the neurotransmitter glutamate (Ekström and Meissl, 2003; Falcón, 1999; Meissl, 1997). The neuroendocrine mode transmits light information through the hormone melatonin. Pineal photoreceptors often have characteristics of both modes, therefore called dual-mode photoreceptors, and the signaling modes may be used in parallel by the cell (Ekström and Meissl, 1997, 2003). Although the main composition of the teleost pineal anatomy is well characterized, information about the different photoreceptor cells and their organization in the pineal organ is inadequately understood. Ventrally to the pineal organ lies a structure that evaginates from the diencephalic roof that is historically referred to as the dorsal sac or saccus dorsalis. In rainbow trout (*Oncorhynchus mykiss*), this structure is described as a folded single-layered epithelial wall that separates the outer meningeal matrix fluid from the inner cerebrospinal fluid (Jansen et al., 1976). Interestingly, this structure is concluded to be the structure that is referred to as the choroid plexus in zebrafish where its role is to produce and regulate components of the cerebrospinal fluid (Henson et al., 2014). The cells of choroid plexus are motile ciliated cells which are important for the cerebrospinal fluid flow (D’Gama et al., 2021). It is also shown that the choroid plexus epithelial cells secrete proteins into the cerebrospinal fluid (Jeong et al., 2024). A possible photoreceptive capacity of the choroid plexus has so far not been investigated.

Evidence for photosensitivity in the teleost pineal organ was earlier described in rainbow trout by microelectrode recordings (Dodt, 1963) and later confirmed histologically by the immunodetection of α-transducin (Ekström et al., 1987). The photoreceptive capacity and sensitivity are dependent on opsins which are retina-bound G-protein coupled receptors (Terakita, 2005), and one of the first identified opsins in the pineal organ of fish was *exorhodopsin* (*exorh*), named “extra-ocular-rhodopsin” due to its similarities to rhodopsin (Mano et al., 1999). *exorh* has also been linked to melatonin synthesis, where loss of exorh through knockdown morpholino studies in zebrafish has been shown to decrease the expression of *exorh* itself and also downregulate the melatonin-synthesizing enzyme *arylalkylamine N-acetyltransferase 2* (*aanat2*) (Pierce et al., 2008). Other opsins have also been identified in the teleost pineal organ. The expression of *vertebrate ancient* (*VA*) *opsin* and the presence of α-transducin, rod-opsin, and cone-opsin immunopositive cells was established in the pineal organ of Atlantic salmon (Philp et al., 2000), and *exorh*- and *melanopsin*-positive cells have been characterized in the Atlantic halibut (*Hippoglossus hippoglossus*) pineal organ (Eilertsen et al., 2014). The expression of *pinopsin*, a green-light-sensitive opsin, has been detected in the caudal parts of the Atlantic tarpon (*Megalops atlanticus*) pineal organ (Fujiyabu et al., 2024) and the expression of *parapinopsin a* (*ppa*) (UV light sensitive) and *parapinopsin b* (*ppb*) (blue light sensitive) has been detected in the rostral parts of the zebrafish pineal organ. Interestingly, *ppb*-positive cells are also expressing the melatonin-synthesizing enzyme *aanat2* and contain serotonin, the precursor of melatonin, indicating that this opsin may play a role in melatonin synthesis (Koyanagi et al., 2015). *Retinal pigment epithelium-derived rhodopsin homolog* (*rrh*) or *peropsin* was first identified in human ocular tissue where a role either as a photoreceptor or a photoisomerase has been suggested. Histology on the mouse eye shows that *peropsin* is present in the microvilli surrounding the photoreceptor outer segment in the retinal pigment epithelium (RPE) (Sun et al., 1997). So far, most in-depth pineal studies have been performed on zebrafish, and few studies have investigated the opsin photoreceptive capacity and cellular organization in Atlantic salmon, a species living in the temperate zone with great seasonal variety.

Melatonin is synthesized in the pineal organ as a direct response to the external light environment and signals “darkness” with low levels during the day and high levels during night (Hardeland et al., 2006). Melatonin is synthesized through several enzymatic steps from the essential amino acid tryptophan. Tryptophan hydroxylase converts tryptophane to 5-hydroxytryptophane which, in turn, is converted to serotonin by aromatic amino acid decarboxylase. Serotonin is converted to N-acetyl-serotonin by Aanat followed by methylation by N-acetylserotonin O-methyltransferase (Asmt) to form melatonin (Axelrod, 1974; Falcón, 1999; Falcón et al., 2010). Although most of the systemic melatonin is produced in the pineal for vertebrates, secondary sources of melatonin are also produced in other cells, tissues, and organs of the body (Claustrat et al., 2005). In addition, melatonin synthesis can also be stimulated by near-infrared light (Tan et al., 2023).

Several components of melatonin synthesis have been described in teleost fish (as reviewed in Falcón et al. (2010) and Saha et al. (2019)). Both serotonin and *N*-acetyl-serotonin were histologically detected by immunohistochemistry in the pike (*Esox lucius*) pineal epithelium (Falcón et al., 1984). *Aanat2* have been detected by *in situ* hybridization in zebrafish (Pierce et al., 2008), and immunolabeling of Asmt was performed on the pineal organ of several fish species, including rainbow trout, showing strong staining in the epithelium close to the pineal lumen (Falcón et al., 1994). In addition, single-cell RNA sequencing of zebrafish pineal photoreceptor cells show that they are *aanat2*- and *asmt*-positive (Zheng et al., 2024). Importantly, Aanat2 is the rate-limiting enzyme in the melatonin synthesis pathway, and in most teleosts the expression of *aanat2* is cycling in a circadian manner with higher levels during the night and lower levels during the day (Saha et al., 2019). Taken together, several studies have described the melatonin-synthesizing features of the teleost pineal organ; however, a histological characterization of *aanat2* and a link between the melatonin-synthesizing factors and the photoreceptive system is missing in Atlantic salmon.

The Atlantic salmon is a species of high commercial interest and a good model for studying rhythmicity as seasonal oscillations in light-driven development and life history transitions. Several studies have analyzed factors regulating these developmental transitions, where an increase in daylength, associated with the transition from winter to spring, initiates the parr–molt transformation, while a decrease in daylength associated with autumn and coming winter initiates sexual maturation (Bjornsson et al., 1994; Hoar, 1988; McCormick et al., 2009; McCormick, 2012; Stefansson et al., 2008). However, there is a lack of insight into how photoreception participates in the regulation of, for example, endocrine pathways. Atlantic salmon have 42 functional genes encoding nonvisual opsins (Eilertsen et al., 2022); however, the complexity of nonvisual photoreception in the pineal organ and which opsin(s) are responsible for the timing and regulation of melatonin synthesis in salmon are still unknown. This study reveals the photoreceptive capacity of the pineal organ of Atlantic salmon, showing that the *exorh*-expressing cells constitute the main photoreceptive elements in the pineal organ and melatonin synthesis is overall linked to this photoreceptor type. A novel finding is that the cerebrospinal fluid producing choroid plexus cells contain photoreceptive elements, suggesting a photoreceptive role in the production and regulation of the cerebrospinal fluid.

## 2 Materials and methods

### 2.1 Ethical statement

Atlantic salmon (*Salmo salar*) parr were obtained from the Industrial and Aquatic Laboratory (ILAB, Bergen, Norway), and the fish were kept until sampling in a facility at High Technology Center, University of Bergen, Norway, given approval by the Norwegian Food Safety Authority (VSID2135). In addition, eggs and sperm from Atlantic salmon were obtained from MOWI (Tveitevågen, Norway), and euthanasia was done by the aquaculture farmers on the field site according to the regulation on slaughterhouses and production facilities for aquaculture animals (FOR-2014-12-15-1831). Fertilization and rearing of the eggs were done in the facilities at the High Technology Center. The fish did not undergo any treatment or handling except euthanasia, and therefore special approval was not required according to Norwegian National legislation via the Norwegian Animal Welfare Act (LOV-2009-06-19) and Regulations on the Use of Animals in Experiments (FOR-2015-06-18-761), given by EU (Directive 2010/63/EU) for animal experiments. A lethal dose of metacaine (MSD Animal Health, The Netherlands) was used to euthanize the fish, on site, before further handling (Ackerman et al., 2005).

### 2.2 Animals and sampling

The fish (Atlantic salmon) were raised under light/dark (LD) cycle of 14:10 from fertilization to first feeding. They were transferred to feeding tanks and reared under LD 20:4 until they reached approximately 11 cm. At this stage, the parr were approximately 1 year old, and they were transitioned to LD 12:12, corresponding to a winter signal. Parr at LD12:12 with weight ranging 25.2–36.4 g were euthanized and sampled for *in situ* hybridization and immunohistochemistry by cardiovascular perfusion with 4% paraformaldehyde. The fish was euthanized by using a lethal dose of MS222 with a concentration of 400 mg/L (Ackerman et al., 2005) under air bubbling conditions. The fish was bled out using a scapula knife to vena cardinalis communis and thereafter perfused with 4% paraformaldehyde. The brains were further processed for cryo-sectioning as described in Norland et al. (2023). The first feeding alevins reared at LD 14:10 (approximately 720 day degrees) were euthanized and fixated in 4% paraformaldehyde for *in situ* hybridization and further processed for cryo-sectioning as described in Eilertsen et al. (2014). For RNA sequencing, two pineal organs from Atlantic salmon parr (StofnFiskur/Benchmark Genetics Iceland) (29.1 and 31.8 g) reared at LD 12:12 were dissected out of the skull after euthanization and snap-frozen in liquid nitrogen before storage in -80°C (sampled at 12:00, ZT4). For molecular cloning, Atlantic salmon parr brain and alevin head and eye were obtained by euthanization, dissection, and snap-freezing in liquid nitrogen before storage in -80°C. All samplings were conducted between 2 and 6 h after onset of light (zeitgeber time: ZT2–ZT6).

### 2.3 RNA extraction and cDNA synthesis

The Atlantic salmon parr pineal organs were added to RNAlater ICE (ThermoFisher Scientific, Waltham, MA, USA) and left for immersion at -20°C for at least 48 h before total RNA isolation using TRI reagent (MilliporeSigma, Burlington, MA, USA) following the manufacturer’s instructions. The two samples of pineal total RNA were pooled, and TURBO DNA-free™ Kit (Thermo Scientific, Waltham, MA, USA) was used to treat the total RNA with DNAase I. RNA integrity number (RIN) was measured to be 9.4 using Agilent2100Bioanalyzer (Agilent Technologies, Santa Clara, CA, USA). Total RNA from the Atlantic salmon parr brain and alevin heads and eyes for molecular cloning was individually isolated and treated with DNase as the pineal organs before cDNA was reversely transcribed using SuperScript III Kit (Invitrogen, Carlsbad, CA, USA) following the manufacturer’s instructions.

### 2.4 RNA sequencing and analyses

The RNA sample of pineal organs was submitted for RNA sequencing at the Genomics Core Facility at the University of Bergen (Norway), and the sample was processed using Illumina TrueSeq Stranded mRNA Sample Preparation Kits (Illumina, Inc., San Diego, CA, USA) according to the manufacturer’s instructions. The sample was sequenced with 75-bp pair-end reads on the Illumina HiSeq4000 sequencing system (Illumina, Inc.). RNA sequencing data were deposited to the European Nucleotide Archive, accession number PRJEB103069. The results were trimmed by Trimmomatic version 0.38 (Bolger et al., 2014) before alignment using STAR version 2.7.0 (Dobin et al., 2013) to the published Atlantic salmon reference genome Index of/pub/release-106/gtf/salmo_salar. Processing of the output files was performed using Samtool version 1.6 (Li et al., 2009), and counts were generated in HTSeq version 0.11.2 (Anders et al., 2015). A normalized count file was generated using DESeq2 version 1.26.0 (Love et al., 2014). The normalized count file was searched for nonvisual opsins (Eilertsen et al., 2022) using revised Ensembl GeneIDs for the nonvisual opsins (**Supplementary Table 1**) due to the update of assembly and gene set from ICSASG_v2 to Ssal_v3.1.

### 2.5 Molecular cloning

Primers for molecular cloning of nonvisual opsins, *aanat2* and *asmt*, were made by *in silico* analyses of the gene sequences in Ensemble (Cunningham et al., 2019) and NCBI (Sayers et al., 2018), and to provide full-length sequences, the primers were placed in the untranslated region or in the start/stop codon for each gene (**Table 1**). A 35-cycle amplification was conducted using Advantage 2 PCR Kit (TaKaRa, Japan). PCR products were extracted from agarose gel using MinElute^®^ Gel Extraction Kit (Qiagen^®^, Germany), and cloning of these PCR products was performed using StrataClone PCR Cloning Kit (Aglient Technologies). QIAprep^®^ Spin Miniprep Kit (Qiagen^®^) was used to purify the plasmids. BigDye™ Terminator v3.1 (Thermo Fisher Scientific) was used to prepare the samples before sequencing, which was performed at the sequencing facility at the University of Bergen (Norway).

**Table 1:** Molecular cloning primers (MilliporeSigma) and probe information.

Target	Ensembl ID	Probe length (bp)	Primer sequence (5′–3′)
*exorh*	*exorh* (**ENSSSAG00000006623**)	1,175	F: ATGAATGGGACGGAGGGCC R: TGCAGTGCAGACAGAGGTAAATTG
*ppa*	*ppa* (**ENSSSAG00000102045**)	1,041	F: ATGGACCACCGACAGCTTC R: TCATTGGGGTGAGACTCGTG
*ppb*	*ppb* (**ENSSSAG00000047145**)	1,138	F: AGAGAGTGGGAGCCTGTTTG R: GTGACACTTTATTGGTGCTGCTG
*tmt1b*	*tmt1b1* (ENSSSAG00000067287) *tmt1b2* (**ENSSSAG00000098456**)	1,219	F: GAAACTAGCCTTCGTGAACAGCAAC R: TGAGCCGTGCATCAACATCC
*tmt3a*	*tmt3a1* (**ENSSSAG00000111843**) *tmt3a2* (ENSSSAG00000108797)	1,107	F: TATTCCCAAAGAGCGCGTAGC R: TCCTCAAGATGCACTAGCAG
*rgra*	*rgra1* (ENSSSAG00000006385) *rgra2* (**ENSSSAG00000048215**)	914	F: TAGAGGCACATCTTTAAGCGAG R: GAACAAGGCAGTTTAACCCAC
*rrh*	*rrh* (**ENSSSAG00000005205**)	1,183	F: TCTTGCTGGTGTAGAAGATTCC R: CACGTATCCCATCCTCTAACAG
*aanat2*	*aanat2.1* (ENSSSAG00000045980) *aanat2.2* (**ENSSSAG00000085086**)	875	F: TCCACAACAGAAGACTGGAAAG R: GATGGGCAGTGCCTTCTCTC
asmt	*asmt.1* (ENSSSAG00000054086) *asmt.2* (**ENSSSAG00000080192**)	1,141	F: GAGGTGTCTAGTGAGAGGCTGAG R: TCTTTGCGACAACAGAGTGATC

For the targets with salmonid-specific autotetraploidization event (Ss4R) paralogs, the Ensembl IDs highlighted in bold were used as template in the probe synthesis (see text for further details).

### 2.6 mRNA probe synthesis

Digoxigenin (DIG) and fluorescein (FITC) antisense and sense probes were prepared according to the manufacturer’s protocol (Roche Diagnostics, Germany). mRNA probe synthesis was performed as described by Thisse and Thisse (2008) using purified PCR product as a template. The synthesized probes were precipitated with LiCl, EtOH, and tRNA (Roche Diagnostics). In the case of Ss4R paralogs, the target region of the probe shared a high sequence similarity (>90% sequence identity), and the probe will hybridize to mRNA of both genes, except for *asmt.1* and *asmt.2*, where it seems like *asmt.1* is a partial gene. Routinely, the sense probes for all genes were used as control to exclude the possibility of unspecific staining.

### 2.7 *In situ* hybridization on sections

*In situ* hybridization (ISH) was performed as described in Norland et al. (2023) and Sandbakken et al. (2012) with a few modifications. Then, 10-µm-thick sections were air-dried before baking at 65°C. Then, ethanol series was used to rehydrate the tissue before washing with 2x SSC buffer (MilliporeSigma). The permeability of the cells was increased by treating the tissue with Proteinase K (MilliporeSigma), followed by post-fixation with 4% paraformaldehyde (MilliporeSigma). The fixative was thoroughly removed using 1x PBS (MilliporeSigma). Triethanolamine and acetic anhydride (MilliporeSigma) were used to reduce background staining. The sections were washed with 2x SSC and rehydrated before mRNA probe hybridization using 4 M urea (MilliporeSigma) instead of formamide in the hybridization mix, and then these were left to hybridize overnight at 65°C. Excessive probes were thoroughly washed away with 2x SSC before treatment with 4 M urea to increase stringency. Partly bound probe was removed by treating the tissue with RNase A (MilliporeSigma). Then, 2x SSC, 0.05% EcoSurf™ EH-9 (MilliporeSigma), and 2% blocking reagent (Roche Dignostics) were used to wash and block the tissue before overnight incubation of DIG secondary antibody (anti-DIG conjugated with alkaline phosphatase, Fab fragments (1:2,000), Roche Diagnostics, RRID: AB_514497) at room temperature. The unbound antibody was washed away with 1x maleate buffer (MilliporeSigma) before NBT/BCIP Ready-to-Use Tablets (MilliporeSigma) was used for visualization. The sections were mounted with 70% glycerol.

### 2.8 Fluorescent double-labeling *in situ* hybridization

Fluorescent double-labeling ISH was performed to identify the expression of two genes together on the same pineal sections to characterize their cellular organization in relation to each other. The protocol was conducted as described in Eilertsen et al. (2014) with a few modifications. The sections are treated the same way as for classic *in situ* hybridization prior to hybridization, where both DIG and FITC probes were applied at the hybridization step and left to hybridize overnight at 65°C. The sections were washed with 2x SSC and treated with 4 M urea and with RNase treatment before visualization. The FITC probes were visualized first by incubating with POD secondary antibody (antifluorescein–horseradish peroxidase (POD), Fab fragments (1:400), Roche Diagnostics, RRID: AB_840257) and then using the TSA Plus Fluorescein System (Akoya Biosciences, Marlborough, MA, USA, catalog no. NEL741001KT). The tissue was blocked in 8% tryptone water (MilliporeSigma) and 0.02% Tween-20 (MilliporeSigma) solution (Kim et al., 2003) for half an hour before the application of the next secondary anti-DIG antibody (anti-DIG conjugated with alkaline phosphatase, Fab fragments (1:2,000), Roche Diagnostics, RRID: AB_514497) and was left for hybridization at room temperature overnight. SIGMAFAST™ FastRed TR/Naphthol AS-MX (MilliporeSigma) tablets were used to visualize the DIG probes. ProLong™ Diamond Antifade Mountant with DAPI (Invitrogen, Thermo Fisher Scientific) was used for section mounting.

### 2.9 Double-labeling using fluorescent *in situ* hybridization and immunohistochemistry on sections

A combination of fluorescent *in situ* hybridization and immunohistochemistry was performed to investigate the presence of cilia by targeting α-tubulin in *peropsin*-positive cells. DIG-labeled probe against *peropsin* (*rrh*) was visualized using SIGMAFAST™ FastRed TR/Naphthol AS-MX (MilliporeSigma) tablets as described above. Mouse anti-α-tubulin antibody (Sigma-Aldrich Cat#T7451, RRID: AB_609894) is a commercially available antibody that targets axons and cilia and has previously been used to label cilia in the choroid plexus of zebrafish (Jeong et al., 2024). The sections were blocked in 2% blocking reagent (Roche Diagnostics) before introducing mouse anti-α-tubulin (1:1,000) (Sigma-Aldrich Cat#T7451, RRID: AB_609894), together with 2x SSC, blocking reagent, and EcoSurf™ EH-9 (MilliporeSigma), for overnight incubation at room temperature. A 45-min incubation with anti-mouse IgG CF™ (Sigma-Aldrich cat. no. SAB4600042, RRID: AB_2532075) was used to visualize the primary antibody, and the sections were mounted using ProLong™ Diamond Antifade Mountant with DAPI (Invitrogen, Thermo Fisher Scientific).

### 2.10 Imaging

Atlantic salmon alevin pineal organs were imaged with ZEISS Axio Scan.Z1 slide scanner (Zeiss, Germany) and ZEN software (Zeiss) using brightfield and ×20 magnification. The parr pineal organs were imaged with Leica K3C and K3M Microscope Camera (Leica Microsystems, Wetzlar, Germany) attached to Leica DM 6000B microscope using Las-X software with brightfield settings (Leica Biosystems, USA). Confocal imaging was performed using laser scanning confocal microscope Olympus FV3000 (Olympus, Japan) and FV31S-SW software (Olympus) with ×10 and ×20 air objectives and ×40 silicone-immersion oil objective (Olympus). The channels used were DAPI for nuclei staining, AF 488 for TSA-stained *exorh*, *ppa*, and *aanat2*, and immunolabeled α-tubulin and AF 555 for FastRed-stained *exorh*, *ppb*, *rgr*, *peropsin*, *aanat2*, and *asmt*. All images were taken with Z scan and, ImageJ 1.53t (ImageJ, National Institutes of Health, USA) was used to generate maximum-intensity z-stack projections. The brightness and contrast adjustments and arrangement of panels for all figures were performed using Adobe Photoshop (San Jose, CA, USA). The co-localization analysis of the expression of *exorh*/*aanat2* and *exorh*/*rgr* was done by using ImageJ/Fiji (version 1.54r) Coloc2 plugins to obtain the 2D intensity histogram, and the Pearson’s *R*-value was calculated. The confocal two-channel images were opened in Fiji and split into separate grayscale images (2,048 × 2,048 pixels); the background was subtracted using a rolling ball (radius 50 pixel) algorithm before running the Coloc 2 procedure (**Supplementary Figure 1**).

## 3 Results

**Figure 1:**
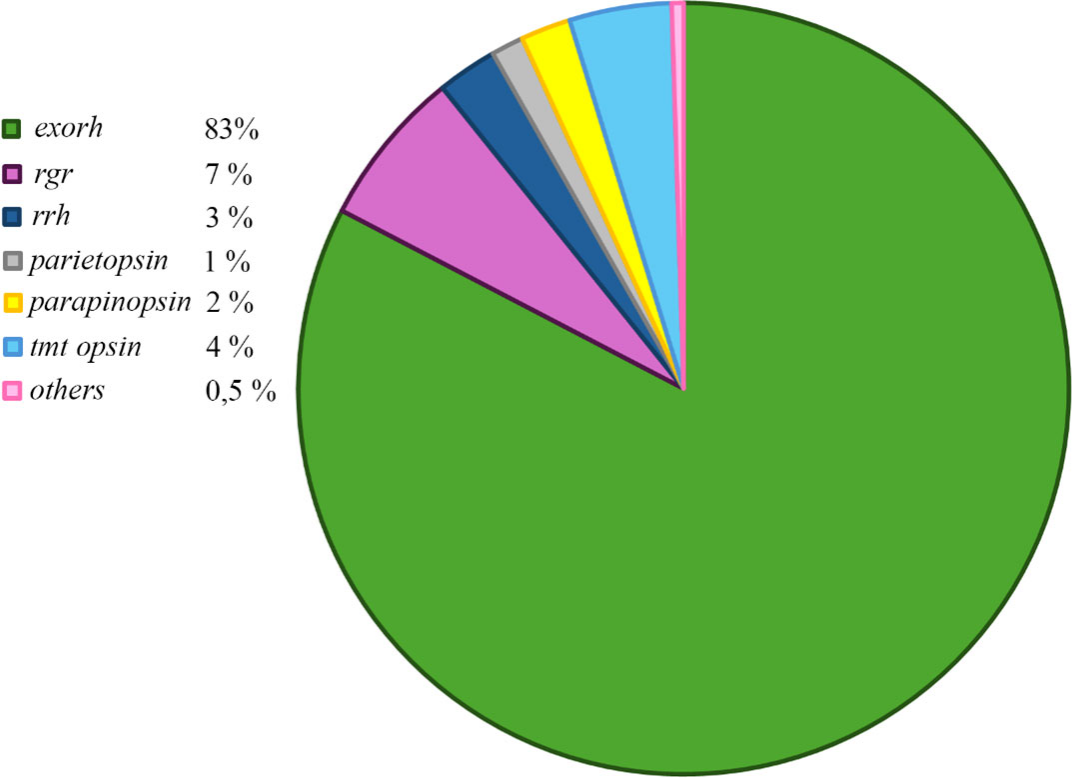
Pie chart of nonvisual opsin mRNA distribution: accounting for 83% of total mRNA counts was *exorhodopsin* (*exorh*). The second highest counts were the group of *retinal G-protein coupled receptor* (*rgr*) *opsins* with 7%. *Teleost multiple tissue* (*tmt*) *opsin* constitutes 4% of the total nonvisual opsin counts. *Peropsin* (*rrh*) has 3% of the total count. The *parapinopsins* (*ppa* and *ppb*) had 2%, and *parietopsin* had 1% of the total nonvisual count, and the rest of the opsins—others have 0.5% of the total nonvisual opsin count. Colors are used to differentiate the opsins and do not relate to their wavelength specificity.

**Figure 2:**
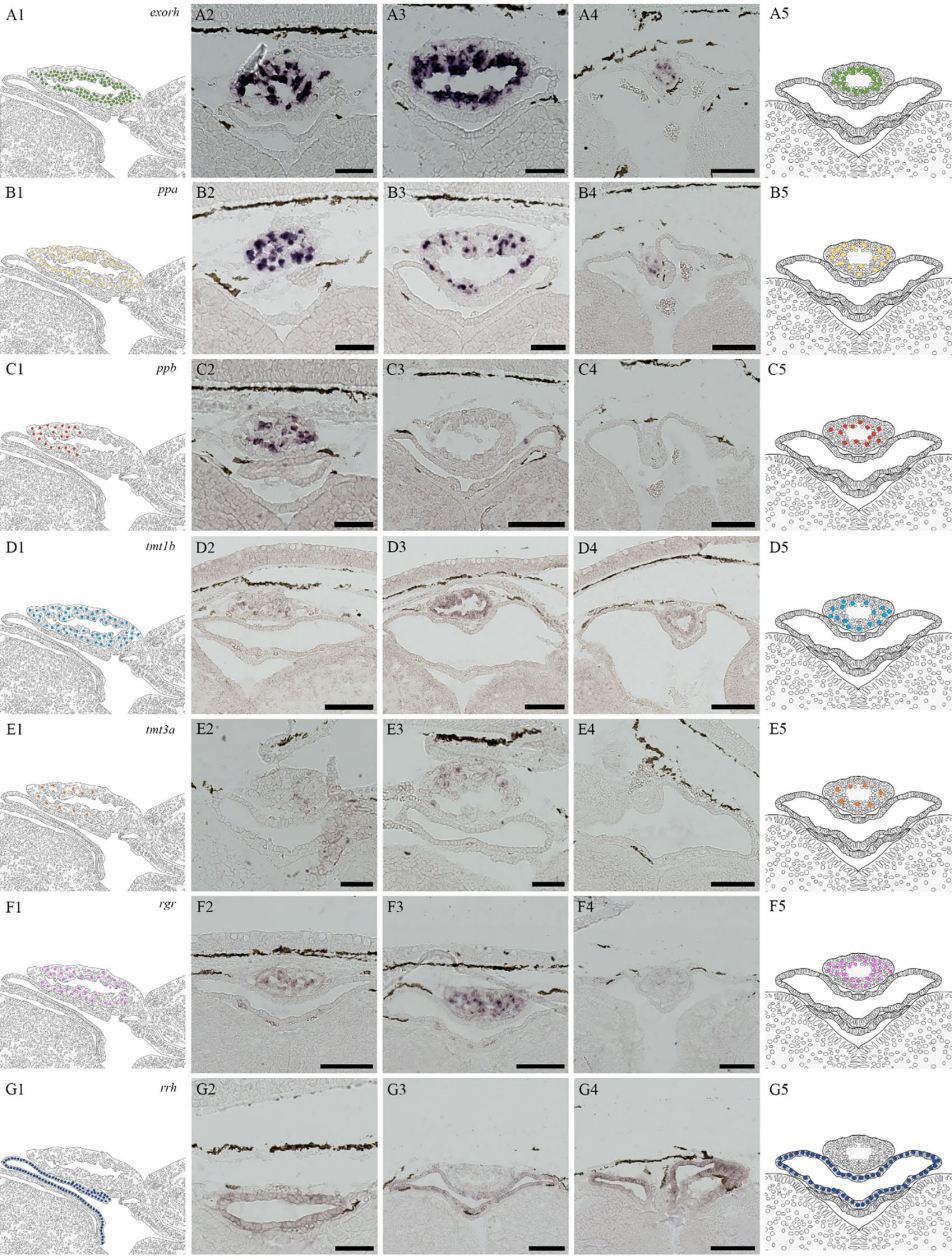
*In situ* hybridization showing the expression pattern of nonvisual opsins on transversal sections of the pineal organ in first-feeding alevin Atlantic salmon. The expression pattern is presented from left to right with one panel for each individual opsin, starting with a sagittal model of the expression **(A1–G1)** and then the rostral **(A2–G2)**, medial **(A3–G3)**, and caudal sections **(A4–G4)**, and finally a transversal model **(A5–G5)** of the expression. **(A1–A5)** *Exorhodopsin* (*exorh*) is expressed in the rostral, medial, and caudal parts of the pineal organ with a very strong expression pattern centered toward the lumen. **(B1–B5)** Expression of *parapinopsin a* (*ppa*) is detected in all parts of the pineal, showing a strong expression defined to specific cells. **(C1–C5)** *Parapinopsin b* (*ppb*) is expressed quite strongly in the rostral parts of the pineal organ, but no expression can be detected in the medial and caudal regions. **(D1–D5)** The expression of *teleost multiple tissue opsin 1b* (*tmt1b1/2*) is scattered dispersedly in all parts of the pineal organ, with a more prominent expression in the medial region. **(E1–E5)** *Teleost multiple tissue opsin 3a* (*tmt3a1/2*) expression can be detected in the rostral and medial parts but not in the caudal regions of the pineal organ. **(F1–F5)** Expression pattern of *retinal G-protein coupled receptor* (*rgra1/2*) in all parts of the pineal organ with a quite dispersed expression pattern. **(G1–G5)** *Peropsin* (*rrh*) is expressed in the rostral, medial, and caudal parts of the dorsal sac located ventrally to the pineal organ. The horizontal dark line in the dorsal region is the pigment epithelium. Axes: **(A1**–**G1)**—top of image is dorsal, bottom is ventral, left is anterior, while right is posterior. **(A2**–**G5)**—top of image is dorsal, bottom is ventral, while left and right are toward the lateral sides. Scale bars: Rostral **(A2–G2)** and medial panels **(A3–G3)** are 50 µm, except from *rrh* medial with 100 µm **(D3)**. Caudal panels **(A4–G4)** are 100 µm.

**Figure 3:**
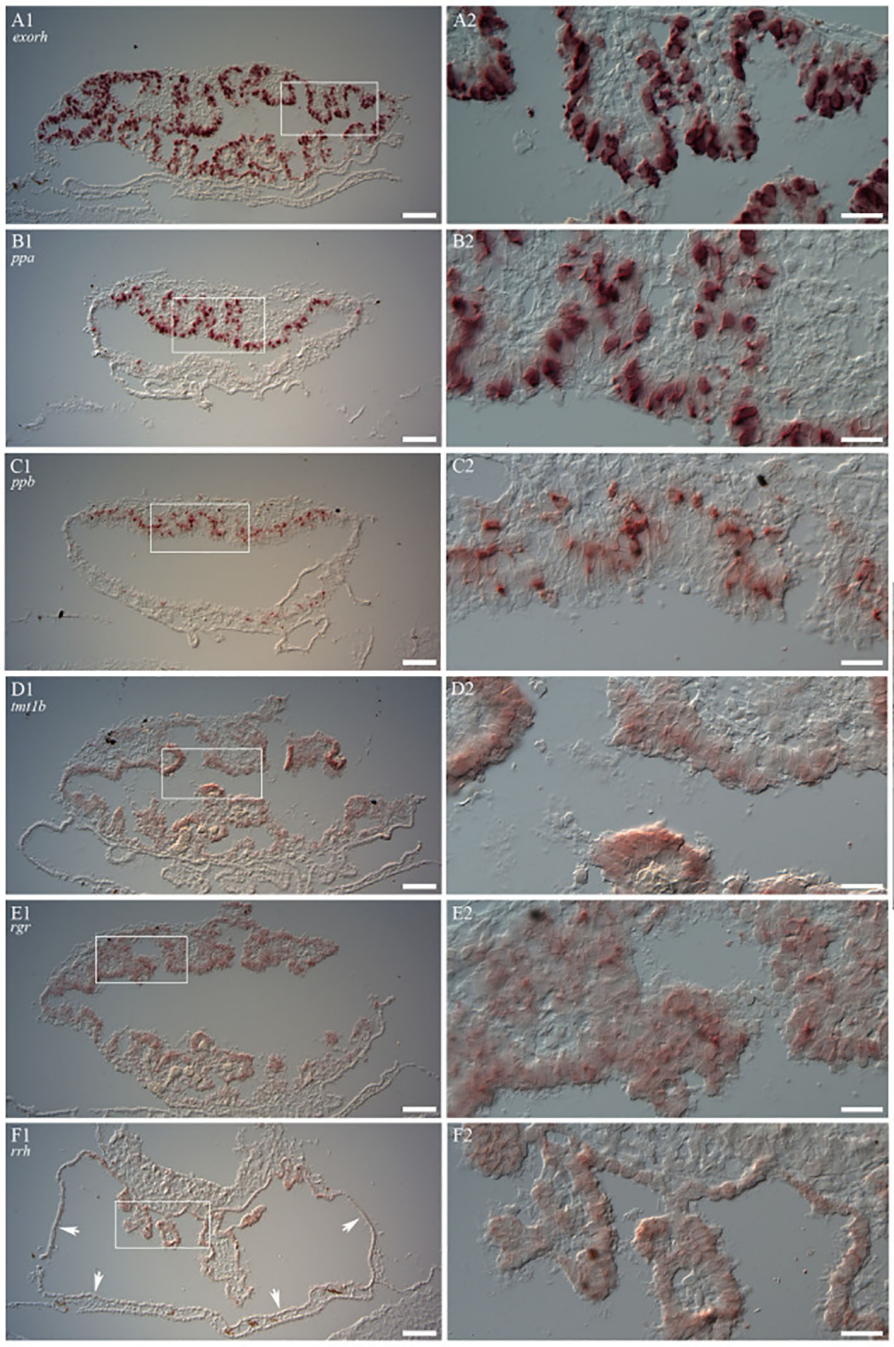
Expression pattern of nonvisual opsins on the transversal sections of the Atlantic salmon parr pineal organ. **(A1, A2)** *Exorhodopsin* (*exorh*) is strongly expressed in the cells located toward the lumen of the pineal. **(B1, B2)** *Parapinopsin a* (*ppa*)-positive cells are located in the more rostral parts of the pineal, and the expression is located toward the pineal lumen. **(C1, C2)** Interestingly, *parapinopsin b* (*ppb*) that is found rostrally in the pineal seems to be located in the second cell layer toward the lumen. **(D1, D2)** *Teleost multiple tissue opsin 1b1/2* (*tmt1b1/2*) is expressed in the cell layer toward the lumen of the cell. **(E1, E2)** Expression of *retinal G-protein coupled receptor* (*rgra1/2*) is found in most parts of the pineal covering several cell layers. **(F1, F2)** *Peropsin* (*rrh*) expression is located in the choroid plexus. Axes: Each image shows dorsal at the top, ventral at the bottom, and lateral sides to the left and right. Scale bars: **(A1, F1)** 100 µm and **(A2, F2)** 25 µm.

**Figure 4:**
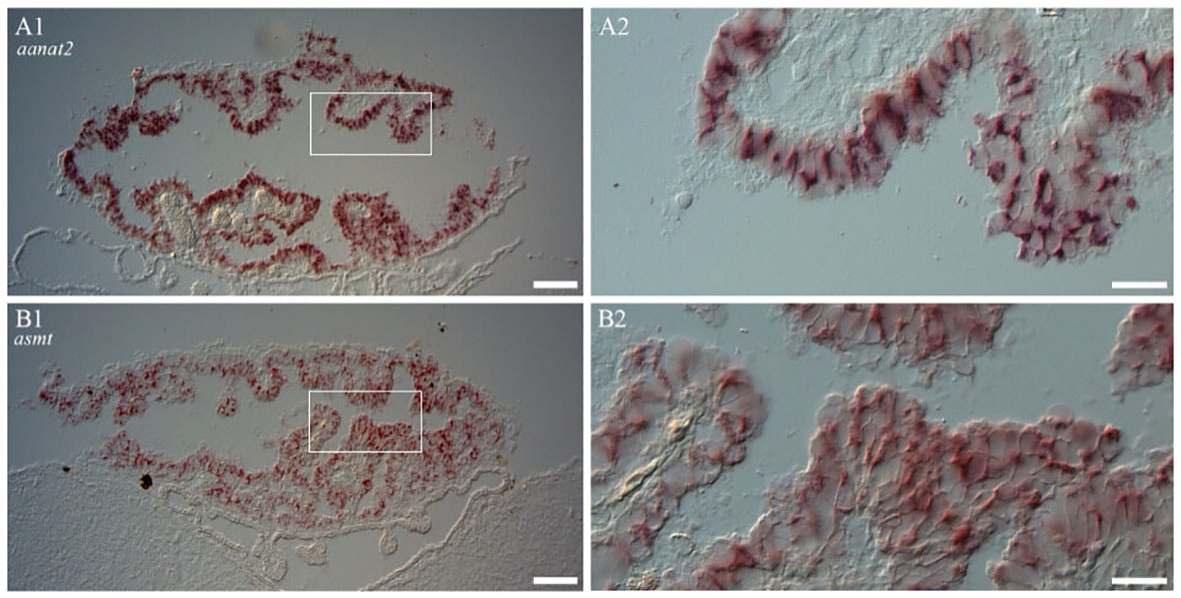
Expression pattern of melatonin-synthesizing enzymes on the transversal sections of the pineal organ in Atlantic salmon parr. **(A1, A2)** The expression of *aanat2.1/2* is quite prominent in the cell layer facing the lumen of the pineal. **(B1, B2)** The *Asmt* expression showed a similar pattern. Axes: Each image shows dorsal at the top, ventral at the bottom, and lateral sides to the left and right. Scale bars: **(A1, B1)** 100 µm and **(A2, B2)** 25 µm.

**Figure 5:**
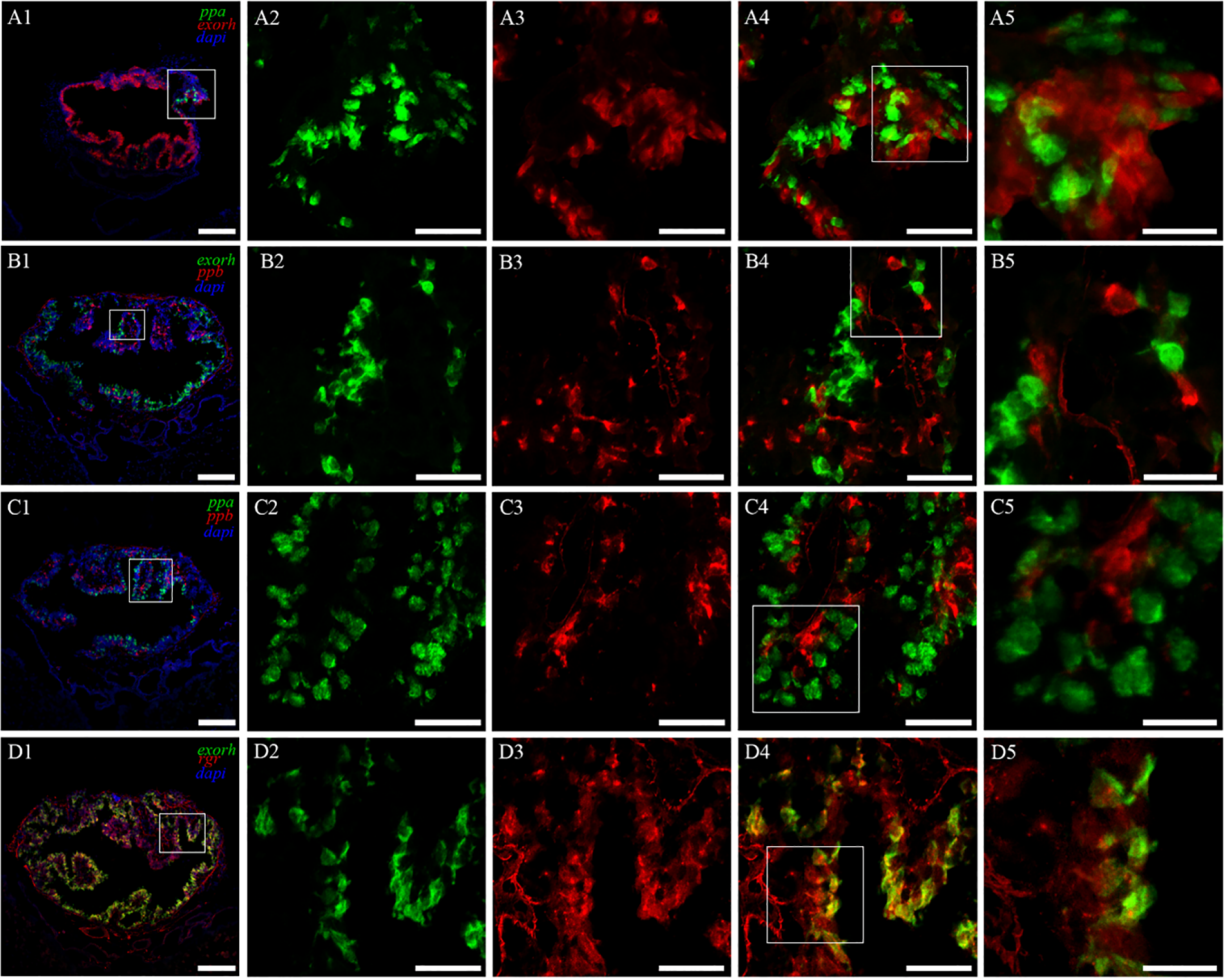
Fluorescent double-labeling *in situ* hybridization of selected nonvisual opsins on the transversal sections of the pineal organ in Atlantic salmon parr. **(A1–A5)** The expression of *parapinopsin a* (*ppa*) and *exorhodopsin* (*exorh*) are in adjacent cells, with no cellular co-localization. **(B1–B5)** The expression of *exorh* and *ppb* clearly displays a cellular distinction between the two opsins. **(C1–C5)** The expression of *ppa* and *ppb* are also clearly in different cells. **(D1–D5)** The expression of *exo* and *rgra1/2* are partly co-expressed. Axes: Each image shows dorsal at the top, ventral at the bottom, and lateral sides to the left and right. Scale bars: **(A1–D1)** 150 µm, **(A2–D4)** 50 µm, and **(A5–D5)** 25 µm.

**Figure 6:**
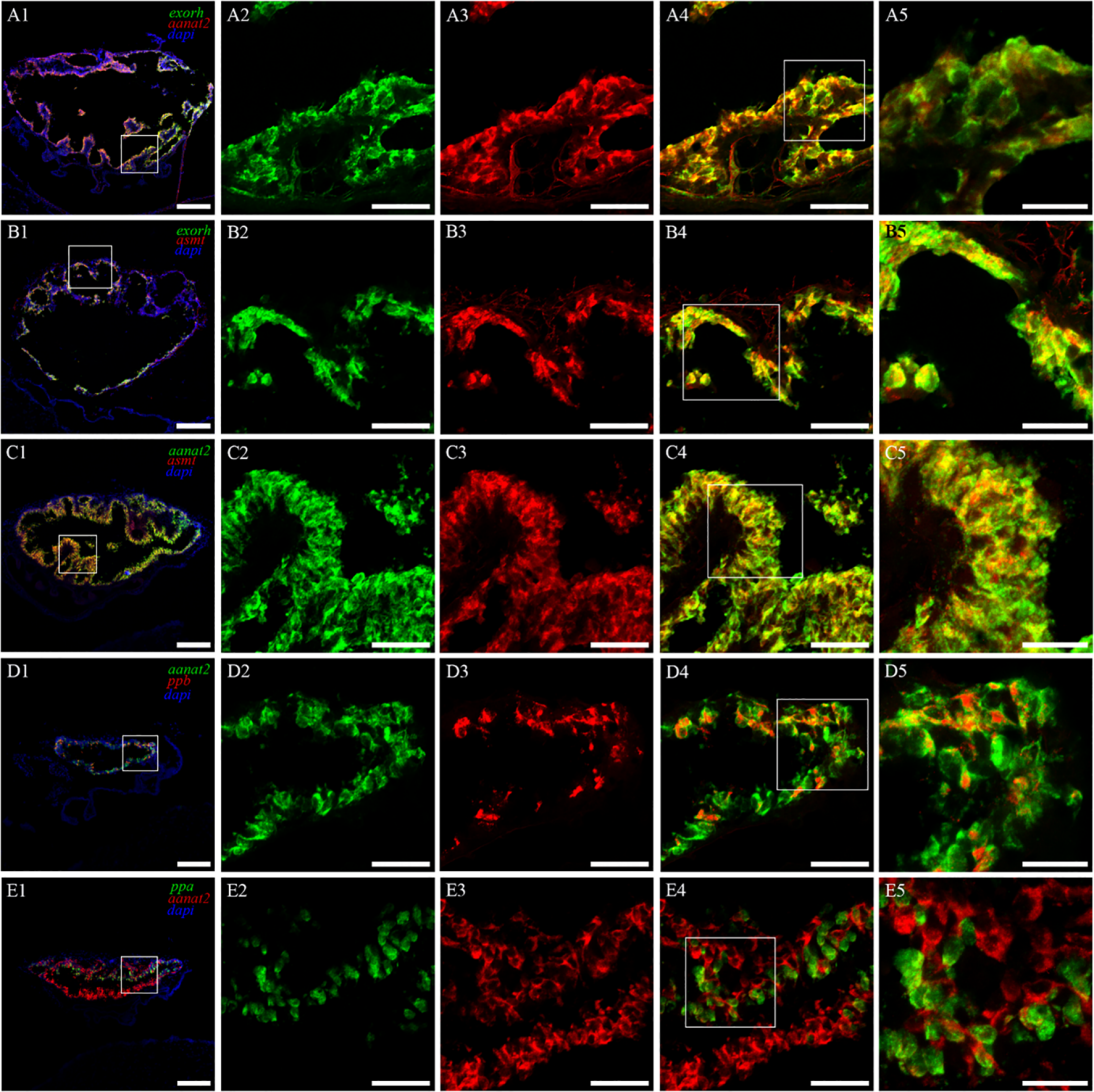
Fluorescent-labeled expression of selected nonvisual opsins and melatonin-synthesizing enzymes on the transversal sections of the pineal organ in Atlantic salmon parr. **(A1–A5)** The expression of *exorh* and *aanat2.1/2* shows a clear cellular colocalization almost covering the entire section. **(B1–B5)** *exorh* and *asmt* are clearly present in the same cells, also covering large parts of the entire section. **(C1–C5)** The expression of the two melatonin-synthesizing enzymes *aanat2.1/2* and *asmt* is clearly colocalized in the same cells. The expression of both genes is widely distributed throughout. **(D1–D5)** The expressions of *aanat2.1/2* and *ppb* were present in the more rostral parts of the pineal, showing not only some cellular colocalization but also some cellular differentiation especially regarding *aanat2.1/2* cells not being *ppb*-positive. **(E1–E5)** The expression of *ppa* and *aanat2.1/2* is very distinct and clearly not colocalized in the same cells. Axes: Each image shows dorsal at the top, ventral at the bottom, and lateral sides to the left and right. Scale bars: **(A1–D1)** 150 µm, **(A2–D4)** 50 µm, and **(A5–D5)** 25 µm.

**Figure 7:**
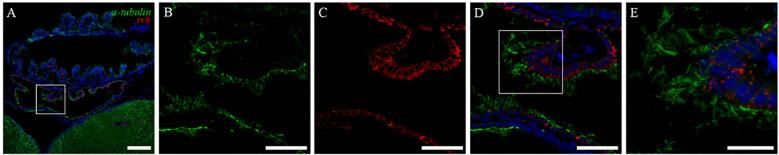
Immunolabeling of α-tubulin and fluorescent *in situ* hybridization of *peropsin* (*rrh*) on the transversal section of the choroid plexus in Atlantic salmon parr. **(A)** Overview of the choroid plexus showing α-tubulin and *rrh* covering the entire choroid plexus epithelial layer. **(B–D)** α-Tubulin labels the cilia of the cell, and *rrh* is expressed in the cell body, almost in a granular pattern. **(E)** A closer view shows the placement of cilia toward the choroid plexus lumen and the *peropsin* expression in the cell body. Axes: Each image shows dorsal at the top, ventral at the bottom, and lateral sides to the left and right. Scale bars: **(A)** 150 µm, **(B–D)** 50 µm, and **(E)** 25 µm.

This study presents the RNA-seq profile of nonvisual opsin in the parr pineal organ of Atlantic salmon (**Figure 1**) that provided the basis of nonvisual opsin *in situ* hybridization in the alevin pineal organ (**Figure 2**) and the parr pineal organ (**Figure 3**). Selecting first-feeding alevin and parr pineal allowed for the opportunity to study the expression pattern both in an early pineal organ, still developing and reliant on yolk-sac nutrient, and a fully developed pineal organ at the parr stage. In addition, the expression pattern of melatonin-synthesizing enzymes, *aanat2* and *asmt*, were characterized by *in situ* hybridization in the parr pineal organ (**Figure 4**). The expression of selected opsins were tested for co-localization using fluorescent double-labeling *in situ* hybridization, showing that most of the opsins are expressed in distinct cells (**Figure 5**). Fluorescent double-labeling *in situ* hybridization shows that *exorh* and *ppb* are co-expressed with the melatonin-synthesizing enzyme *aanat2* (**Figure 6**). A novel finding shows that *peropsin* (*rrh*) is expressed in the Atlantic salmon ciliated choroid plexus cells (**Figure 7**).

### 3.1 RNA seq profile of nonvisual opsins in the Atlantic salmon parr pineal organ

The normalized count file generated by RNA sequencing profile of Atlantic salmon parr pineal organ showed that several nonvisual opsins are present. The dominating opsin is definitely *exorhodopsin* (*exorh*) with 83% of the total nonvisual opsin count (**Figure 1**), followed by the *retinal G-protein coupled receptor* (*rgr*) genes with 7% of the total count.

### 3.2 Opsin expression pattern in the pineal organ of a first-feeding Atlantic salmon alevin

Based on the RNA sequencing results and screening of nonvisual opsins in the brain, the expression pattern of selected nonvisual opsins was characterized in the pineal organ of first-feeding Atlantic salmon (**Figure 2**). The results show that *exorhodopsin* (*exorh*) is the major opsin being expressed in the cells closest to the lumen, covering the entire rostral–caudal region (**Figure 2A**). The expression of *ppa* spans the rostral–caudal axis in the alevin pineal organ with a scattered expression pattern. The expression is most prominent in the rostral regions and more scattered in the caudal part (**Figure 2B**). *Parapinopsin b* (*ppb*) was only found to be expressed in the rostral parts of the pineal organ (**Figure 2C**). The *teleost multiple tissue opsin* (*tmt1b1/2*) had a less prominent expression pattern, with the most prominent expression in the medial regions (**Figure 2D**), and *teleost multiple tissue opsin 3a* (*tmt3a1/2*) also had a less prominent expression, located in rostral and medial parts (**Figure 2E**). *Retinal G-protein coupled receptor* (*rgra1/2*) was quite prominently expressed in all regions of the pineal organ (**Figure 2F**). *Peropsin* (*rrh*) showed no expression in the pineal organ but was expressed in the more ventral choroid plexus (**Figure 2G**).

### 3.3 Nonvisual opsin expression pattern in the pineal organ of the Atlantic salmon parr

Comparing the alevin pineal organ in **Figure 2** to the parr pineal organ in **Figure 3**, it is shown that the pineal epithelium has changed from what seems like a simple circle of cells surrounding the lumen to a larger and highly folded epithelium. *exorh* is the major opsin expressed in the pineal epithelium, covering the entire cell layer close to the lumen (**Figure 3A**). *Ppa* was expressed both in the dorsal and ventral part of the most rostral section; however, further in the caudal direction where the pineal lumen separates the dorsal and the ventral part, *ppa* was only expressed dorsally, in cells closest to the lumen (**Figure 3B**). A similar expression pattern was detected for *ppb*; but differently to the *ppa*-positive cells that were confined to the cell layer closest to the pineal lumen, the *ppb*-positive cells were detected in the second cell layer (**Figure 3C**). *Tmt1b1/2* expression can be found in the cell layer toward the lumen of the pineal organ (**Figure 3D**). The expression of *rgra1/2* covered most parts of the pineal epithelium, including the cell layer closest to the lumen (**Figure 3E**). The expression of *rrh* was not detected in the pineal epithelium; however, it was prominently expressed in the choroid plexus ventrally to the pineal organ (**Figure 3F**).

### 3.4 Expression of melatonin-synthesizing enzymes in the pineal organ of the Atlantic salmon parr

The expression pattern of the melatonin-synthesizing enzymes *aanat2.1/2* and *asmt* was analyzed by *in situ* hybridization in the parr pineal organ (**Figure 4**). *Aanat2.1/2*, the rate-limiting enzyme of melatonin synthesis was expressed quite strongly in the cell layer facing toward the lumen throughout the entire pineal organ (**Figure 4A**). The last enzyme in melatonin synthesis, *asmt*, was found to have a similar pattern (**Figure 4B**).

### 3.5 Double fluorescent *in situ* hybridization of nonvisual opsins in the Atlantic salmon parr pineal organ

Double fluorescent *in situ* hybridization was done to reveal the presence or absence of cellular colocalization between nonvisual opsins in the pineal organ (**Figure 5**). The results showed that *ppa* and *exorh* clearly had no cellular co-localization (**Figure 5A**). Moreover, *exorh* and *ppb* had a clear cellular distinction in their expression pattern (**Figure 5B**). The two paralogs of *parapinopsin* (*ppa* and *ppb*) were also not present in the same cells (**Figure 5C**). Lastly, the expression of *exorh* and *rgra1/2* was partly co-expressed (**Figure 5D**).

### 3.6 Investigation of a potential colocalization of selected opsins and melatonin-synthesizing enzymes in the Atlantic salmon parr pineal organ

Double FISH was used to investigate possible cellular co-localization between selected opsins and melatonin-synthesizing enzymes in the salmon parr pineal organ (**Figure 6**). The expression of the melatonin-synthesizing enzymes *aanat2.1/2* and *asmt* was both found in the outermost cell layer closest to the lumen covering the entire rostral to caudal gradient. This indicates the presence of melatonin synthesis in the major parts of the pineal organ. The expression of *exorh* and *aanat2.1/2* was present in the same cells (**Figure 6A**), as well as *exorh* and *asmt* (**Figure 6B**). *Aanat21/2* and *asmt* were also expressed in the same cells (**Figure 6C**). The expression of *aanat2.1/2* and *ppb* was co-localized in some of the same cells, but there were *aanat2.1/2*-positive cells that were not *ppb*-positive (**Figure 6D**). The expression of *ppa* and *aanat2.1/2* were clearly distinctive with no cellular co-localization (**Figure 6E**).

### 3.7 *Peropsin* is expressed in ciliated choroid plexus cells

Fluorescent *in situ* hybridization shows the expression of *peropsin* in choroid plexus cells where cilia is immunolabeled with α-tubulin (**Figure 7**). The cilia extended into the lumen, while *peropsin* expression is clearly present in the epithelial cell body.

## 4 Discussion

This study presents a comprehensive mapping of nonvisual photoreceptors in the pineal organ and choroid plexus of Atlantic salmon. The results indicate that *exorh* is the main opsin regulating melatonin synthesis in salmon, with the co-expression of *exorh* and genes coding for the important enzymatic steps of melatonin synthesis. The co-expression of *exorh*, *aanat2.1/2*, and *asmt* covers the entire rostral–caudal gradient of the pineal organ, and the expression is in the cell layer closest to the pineal lumen. Furthermore, there is a co-expression of *ppb* and *aanat2.1/2* in the rostral part of the pineal organ. These results reveal that the gene expression of melatonin-synthesizing enzymes is present in photoreceptor cells in the major parts of the pineal organ. A novel finding is the detection of opsin in the cells of the choroid plexus where the one-cell-layer-thick epithelium expresses *peropsin* and has cilia stretching out in the lumen.

### 4.1 Development of the pineal organ to a folded epithelium of cells surrounding the pineal lumen

Several studies have analyzed the histological structure and photoreceptive elements of the pineal organ of teleosts (Blackshaw and Snyder, 1997; Eilertsen et al., 2014; Ekström et al., 1987; Fujiyabu et al., 2024; Koyanagi et al., 2015; Mano et al., 1999; Philp et al., 2000; Pierce et al., 2008). Here the analyses of photoreceptor cell distribution in Atlantic salmon at the alevin and parr stage revealed that the pineal organ goes through comprehensive growth and development. The pineal organ at the alevin stage is a tube-like structure with a central lumen that develops to a larger structure with a highly folded epithelium. The pineal lumen is an extension of the third ventricle of the brain (Ekström and Meissl, 1997), and the folded epithelium allows more cell surfaces to contact the cerebrospinal fluid filling the lumen. In addition, more cells will potentially lead to a higher responsiveness to light and more synthesis of melatonin in the dark. The cellular organization of the pineal organ constitutes photoreceptor cells arranged with their outer segment directed toward the lumen.

### 4.2 Photoreceptive pineal organ of Atlantic salmon

RNA sequencing of the pineal organ of Atlantic salmon parr revealed that *exorhodopsin* is the nonvisual opsin with the strongest expression, consisting of around 80% of the total RNA-seq counts for nonvisual opsins. This result is consistent with studies in zebrafish showing that *exorh* is strongly expressed in the pineal organ at both early developmental stages (Eilertsen et al., 2014; Noche et al., 2011; Pierce et al., 2008) and in the adult pineal organ (Davies et al., 2015; Mano et al., 1999). Furthermore, single-cell RNA sequencing of the zebrafish pineal organ shows that *exorhodopsin* is highly expressed in the abundant rod-like cells, while cone-like cells with *opn1lw1* expression only make up a small fraction of the photoreceptor cells (Zheng et al., 2024). The second highest count among the nonvisual opsins in the parr pineal organ was a group of the genes in the *retinal G-protein coupled receptor* (*rgr*) *opsin* class with 7% of the total nonvisual opsin count. The pineal expression of *rgr* has previously been described in the chick pineal organ (Bailey and Cassone, 2004), and single-cell RNA sequencing of the zebrafish pineal organ also revealed the presence of *rgr* in a cell type classified as retinal pigment epithelium-like cells (Zheng et al., 2024). *Teleost multiple tissue* (*tmt*) *opsins* account for 4% of the total pineal nonvisual opsin count from RNA sequencing, and the expression is in consistence with other teleosts, as the expression of genes in the *tmt* class of opsins has been found to be present in the medaka pineal organ (Fischer et al., 2013; Sato et al., 2021) and in the adult zebrafish pineal organ (Davies et al., 2015). Furthermore, p*eropsin* (*rrh*) constitutes 3% of the total nonvisual RNA count, and *peropsin* has also been described in the chick pineal organ (Bailey and Cassone, 2004), and the gene expression profile of adult zebrafish pineal organ shows a significant expression of *peropsin* (Davies et al., 2015). The results revealed that *parapinopsin* (*ppa* and *ppb*) constitutes 2% of the total pineal nonvisual count, and these results are in agreement with the gene expression profile for *ppa* and *ppb* in zebrafish (Davies et al., 2015). Taken together, the transcriptomic profile of salmon parr pineal organ reveals that several opsins are expressed as in other teleosts where *exorh* emerges as the main photoreceptive element.

### 4.3 Most pineal nonvisual opsins are expressed in the cell layer closest to the pineal lumen

The expression studies show that *exorh* is expressed in the entire rostral–caudal region of the pineal organ, in the cell layer close to the pineal lumen. At the alevin stage, the expression is present in several cell layers, but the expression is confined to the lumen of the pineal organ. However, in the parr pineal organ, the expression is defined to the epithelium layer that is closest to the lumen. This expression pattern is in agreement with *in situ* hybridization targeting *exorhodopsin* and immunostaining against bovine rhodopsin in the adult zebrafish pineal organ (Mano et al., 1999) and at the earlier developmental stages of zebrafish (Noche et al., 2011; Pierce et al., 2008) and Atlantic halibut (Eilertsen et al., 2014). The two *parapinopsins*, *ppa* and *ppb*, in salmon were expressed in the pineal organ at the alevin stage. The expression of *ppa* was found in the entire rostral–caudal region, with the most prominent expression in the rostral cells, while *ppb* was only expressed in the rostral part of the pineal organ. *Ppa* has been described in catfish, with expression in the majority of the parapinealocytes (hence its name), cells of the pineal stalk, a subset of pineal photoreceptor cells, and the lateral habenula (Blackshaw and Snyder, 1997). Furthermore, the UV-light-sensitive *ppa* opsin and the blue-light-sensitive *ppb* opsin were shown to be located in the rostral areas of the pineal organ in both zebrafish and rainbow trout (Koyanagi et al., 2015), which is also the case for the Atlantic salmon pineal organ. There is also a clear dominating presence of *ppa* and *ppb* in the dorsal regions in the rostral sections of the Atlantic salmon pineal organ, which interestingly is also described for parapinopsin in lamprey (*Lethenteron japonica*) (Koyanagi et al., 2004). Intriguingly, we find *ppb* to be expressed in the second closest cell layer of the pineal lumen and not the closest cell layer as for the other opsins described. This expression indicates that *ppb* is localized in secondary neurons underneath the primary photoreceptor cells located toward the lumen in a similar organization as seen in the retina where nonvisual opsins like *melanopsin*- and *VA opsin*-positive cells are found in the different nuclear layers (Sandbakken et al., 2012). At the alevin stage, *tmt1b1/2* is expressed in the entire pineal organ with a more prominent expression in the medial region, while *tmt3a1/2* is only expressed in the rostral and medial parts with the highest expression in medial regions. At the parr stage, pineal *tmt1b1/2* expression was confined to the epithelial layer closest to the pineal lumen, while *tmt3a1/2* was not detected. The expression of *tmt opsin* has been described to be present in several tissues in zebrafish, including the brain (Moutsaki et al., 2003) and in the retina and brain of medaka, with a strong expression both in the deep brain and in the pineal organ (Fischer et al., 2013; Sato et al., 2021). *Retinal G-protein coupled receptor* (*rgra1/2*) opsin has a broad expression pattern covering the entire rostral–caudal gradient of the pineal organ. Expression is present in several cell layers and not just one defined cell layer as seen for the other opsins investigated. The expression is prominent in both the alevin and parr pineal organ. The expression of *rgr* has previously been described in the chick pineal organ, also here covering most of the entirety of the pineal organ (Bailey and Cassone, 2004). Unexpectedly, we detected *peropsin* (*rrh*) expression in the choroid plexus located ventrally to the pineal organ in both the alevin and the parr stage brain, and the expression covers the entirety of the choroid plexus extending the complete rostral–caudal gradient. The expression of *rrh* has also been described in chicken, but in the pineal organ itself and not in the choroid plexus (Bailey and Cassone, 2004).

### 4.4 Cellular organization of photoreceptors

For a detailed description of the photoreceptive cellular organization in the pineal organ, double-labeling fluorescent *in situ* hybridization on selected nonvisual opsins was performed. Interestingly, a clear cellular distinction of the nonvisual opsin expression in individual cells was shown, indicating that the photoreceptive capacity of the pineal organ builds on the complexity of single-opsin-expressing photoreceptors that may allow a wider sensitivity of different wavelengths of light. The results showed that *ppa*, *ppb*, and *exorh* expressions were clearly confined to different cells with no overlap and confirmed the distinct expression of the two parapinopsins as seen in other teleosts (Koyanagi et al., 2015). In contrast, some studies find that different opsin classes are present in the same photoreceptor cell. In Atlantic halibut, a cluster of cells in the hindbrain, associated with hatching, were positive for both *VA opsin* and *melanopsin* (Eilertsen et al., 2018), and in the parietal eye of lizard parietopsin and pinopsin were found in the same cells (Solessio and Engbretson, 1993; Su et al., 2006).

**Figure 8:**
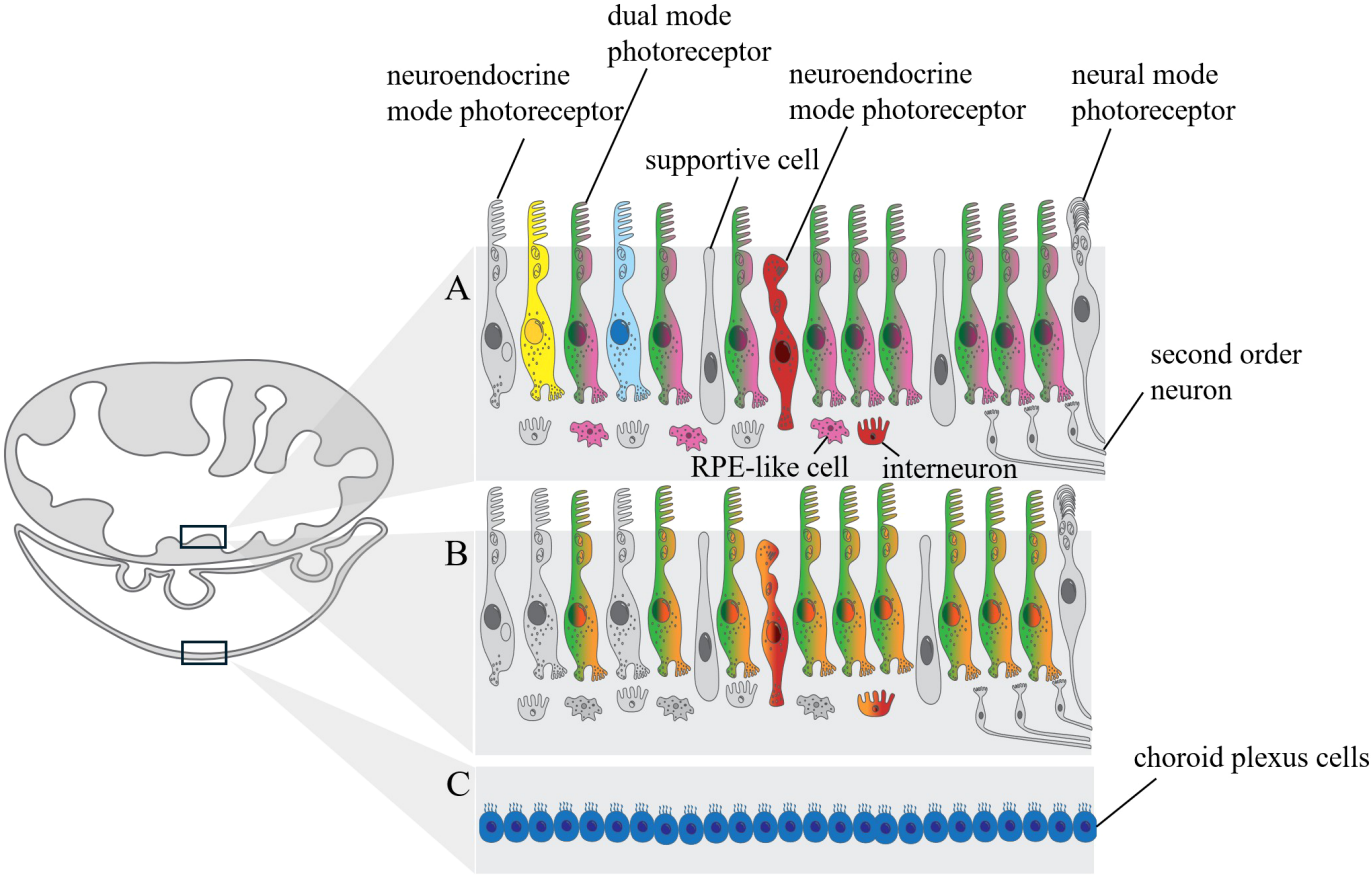
A model for cell location and distribution of expression patterns of the different opsins and melatonin-synthesizing enzymes in the parr pineal based on *in situ* hybridization and RNA sequencing data. **(A)** Hypothetical cellular distribution of the different opsins found expressed in the pineal. The expression of *ppa* (yellow) is hypothesized to be in dual-mode pineal photoreceptors. The expression pattern of *ppb* (red) is more internalized, seemingly a cell row away from the pineal lumen, and is hypothesized to be expressed either in dual-mode photoreceptors cells with less developed outer segments and/or in interneurons. Note that both *ppa* and *ppb* are expressed only in the rostral areas of the pineal. The expression of *exorh* (green) is hypothesized to be located in dual-mode photoreceptors due to its co-localization with *aanat2* and *asmt*. *Tmt1b*-positive cells are located in the same cell layer toward the lumen similar to photoreceptors expressing *aanat2* and *asmt*. However, further studies are needed to verify if these photoreceptors express both *tmt1b* and melatonin-synthesizing enzymes. **(B)** Expression of *aanat2* (orange) co-localized with the opsins *exorh* (green) and *ppb* (red). **(C)** Expression of *peropsin* (dark blue) in the choroid plexus cells. Illustration modified from Ekström and Meissl (1997) and Falcón and Muñoz-Cueto (2024). Colors are used to differentiate the opsins and do not relate to their wavelength specificity.

The expression of *rgra1/2* covered several cell layers of the pineal organ both at the alevin and parr stage and was shown to be partly co-expressed with *exorh*. The intracellular co-localization of the two genes might be an example of dual photoreceptor capacity in these cells. However, it is suggested that Rgr is a photoisomease, and in zebrafish pineal organ the expression of *rgr* was confined to retinal pigment epithelium-like cells (Zheng et al., 2024). In mice, RGR has been shown to regenerate the chromophore from the all-*trans* to the 11-*cis* isomer to maintain photosensitivity (Chen et al., 2001), and in the bovine retinal pigment epithelium, RGR together with cellular retinaldehyde-binding protein has been shown to mediate the regeneration of the chromophore (Zhang et al., 2019). Notably, the expression of *rgr* is partly present in the same cells as *exorh* in the pineal organ, while in the retina, Rgr is present in the retinal pigment epithelium and not the photoreceptor cells itself (Chen et al., 2001). This may suggest that the pineal photoreceptor has the components needed for chromophore regeneration within itself and that it is not solely dependent on other cells for this process as is the case for photoreceptors in the retina. A model for the hypothetical cellular location of opsin expressions in the parr pineal organ is presented in a schematic drawing in **Figure 8**.

### 4.5 Exorhodopsin (and ppb) may drive melatonin synthesis

The important enzymes of melatonin synthesis, *aanat2.1/2* and *asmt*, were found to be quite extensively expressed and covered most of the entirety in the salmon pineal organ. These results are in line with *aanat2* expression in zebrafish (Pierce et al., 2008) and immunolabeling of Asmt in rainbow trout (Falcón et al., 1994). In addition, single-cell RNA sequencing has shown that *aanat2.1/2* and *asmt* are expressed in the photoreceptor cells of the pineal organ in zebrafish (Zheng et al., 2024). Therefore, double fluorescent *in situ* hybridization was performed to investigate the photoreceptive regulation of melatonin synthesis in the salmon pineal organ. The results show that *exorh* is present in the same cells as the melatonin-synthesizing enzymes *aanat2.1/2* and *asmt*, suggesting that *exorh* is the major photoreceptive player of melatonin synthesis. However, whether *exorh* performs a direct light regulation of melatonin synthesis or whether synthesis is regulated by a molecular clock system controlling the expression of *aanat2* is not clear. The Aanat2 paralogs with complete E-boxes in salmon (McStay et al., 2014) were recently shown to be cyclic with an acrophase in the transition between light and dark (Eilertsen et al., 2024). However, the melatonin level in salmonids, in contrast to other teleosts, lacks rhythmicity upon switching from the LD cycles to constant darkness (Gern and Greenhouse, 1988; Iigo et al., 2007; Thibault et al., 1993). A study using morpholino to knock down *exorh* found that zebrafish embryos injected with morpholinos had a significantly reduced expression of both *exorh* and *aanat2*, indicating that *exorh* regulated the transcription of *aanat2* and ultimately melatonin production (Pierce et al., 2008). To add further evidence that *exorh* plays a role in regulating melatonin synthesis, the spectral specificity of *exorh* in zebrafish is approximately 498 nm (Tarttelin et al., 2011) within the wavelength range of 512 ± 10–15 nm, which is most effective in suppressing melatonin levels (Ziv et al., 2007).

Furthermore, in zebrafish, *ppb* and not *ppa* has been suggested to have a role in melatonin synthesis due to its colocalization with *aanat2* and serotonin (Koyanagi et al., 2015). In this study, a co-localization of *ppb* and *aanat2.1/2*, and not *ppa*, was also found in the Atlantic salmon pineal organ, and this is in line with the indications of functional differentiation among the parapinopsins (Koyanagi et al., 2017). However, both the expression pattern and the distribution of *ppb*-positive photoreceptors in the pineal organ is far less extensive, with a confined expression pattern to the rostralmost part of the pineal organ as opposed to the extensive expression of *exorh*-positive photoreceptors covering the entirety of the rostral–caudal axis of the pineal organ. This can indicate that the photoreceptive activity of *ppb* has a supportive role rather than a leading role in the photoreceptive regulation of melatonin synthesis in Atlantic salmon. *exorh* and *ppb* were found to be co-expressed with *aanat2* (**Figure 6**), which strongly indicates a role in the synthesis of melatonin. A functional link to the other opsins studied—*ppa*, *tmt1b*, and *tmt3a*—have not been verified in this study. In general, there is a lack of specific information on the functional role of nonvisual opsins (Perez et al., 2019). However, analyses of the teleost retina show that nonvisual opsins are expressed in retinal interneurons (Matos-Cruz et al., 2011). Interneurons are involved in the retinal processing of light information (Connaughton, 2011), and perhaps other opsins, like *ppb* in the pineal organ, have such function. Seasonal changes in light environment is a very important cue in the correct timing of salmon development (Bjornsson et al., 1994; Hoar, 1988; McCormick et al., 2009; McCormick, 2012; Stefansson et al., 2008), and the need for different photoreceptors may change based on the time of day, season, and whether the fish is in a freshwater or seawater environment. The multi-opsin characteristics of the pineal organ can be hypothesized to allow a more broad-spectrum perception of the light environment, permitting a more fine-tuned sync of the internal physiological response to the external environment.

### 4.6 Photoreception in choroid plexus cells

In this study, expression of *peropsin* (*rrh*) was found in the choroid plexus of the Atlantic salmon both at the alevin and parr stage. A study performed on *Rrh* -/- mice indicate that peropsin controls vitamin A storge in RPE and/or the movement of vitamin A from the retina to the RPE (Cook et al., 2017). In spiders, peropsin has been suggested to be a dark-active, light-inactivated type of opsin (Nagata et al., 2018). The choroid plexus produces and regulates the components of the cerebrospinal fluid (Henson et al., 2014). In zebrafish, the choroid plexus cells were found to be motile ciliated cells with importance for cerebrospinal fluid flow (D’Gama et al., 2021). Immunostaining with antibody against α-tubulin in Atlantic salmon parr revealed the presence of cilia in choroid plexus cells. A combination of *in situ* hybridization and immunohistochemistry further showed that the choroid plexus cells expressing *peropsin* have cilia; this may indicate a photic regulatory mechanism in this cell type that produces the cerebrospinal fluid of the brain.

Interestingly, several melatonin-synthesizing enzymes have been shown to be present in the rat choroid plexus, including AANAT, providing evidence for the synthesis of melatonin in the choroid plexus (Quintela et al., 2018). It is interesting to hypothesize if this is also the case in fish; however, there was no staining found of *aanat2* or *asmt* in the choroid plexus in this study.

## 5 Concluding remarks

The opsin expression patterns presented in this study clearly indicate that the pineal organ of Atlantic salmon is multi-photoreceptive, dominated by a photoreceptor cell type expressing *exorhodopsin*. Except for the *rgr* opsin that seems to be co-expressed together with other opsins and may function as a photoisomerase, the results show that the pineal organ of Atlantic salmon constitutes single-opsin-class-expressing photoreceptor cells. The majority of the photoreceptors are located toward the pineal lumen and, as in the retina, probably function as the primary photoreceptive element, except for *ppb* which seems to be found in the deeper cell layer and may have a modulating function. The novel finding of *peropsin* in the choroid plexus opens the possibility of a direct light regulation of cerebrospinal fluid physiology.

## Data Availability

The datasets presented in this study can be found in online repositories. The names of the repository/repositories and accession number(s) can be found below: https://www.ebi.ac.uk/ena, PRJEB103069.

## Supplementary material

FIGURE S1 Co-expression analysis of exorh-aanat2.1/2 and exorh-rgr in the pineal organ in Atlantic salmon parr. A: Expression of exorh shown in gray scale of confocal image, of double ISH labeling (green channel in Figure 6A1). B: Expression of aanat2.1/2 shown in gray scale (red channel in Figure 6A1). C: 2D pixel intensity histogram, (x-axis is green channel and y-axis is red channel) showing high degree of pixel overlap between the two channels, Pearson’s R value 0,85. D: Expression of exorh shown in gray scale of confocal image, of double ISH labeling (green channel in Figure 5D1). E: Expression of rgr shown in gray scale (red channel in Figure 5D1). F: 2D pixel intensity histogram, (x-axis is rgr and y-axis is exorh) showing some degree of pixel overlap between the two channels, Pearson’s R value 0,64.
