# Supplementary figures and images for: The photoreceptive and neuroendocrine pineal organ of Atlantic salmon
Source: Front Physiol. 2026 May 25;17:1778109. doi: 10.3389/fphys.2026.1778109 (PMC13243063; doi:10.3389/fphys.2026.1778109)

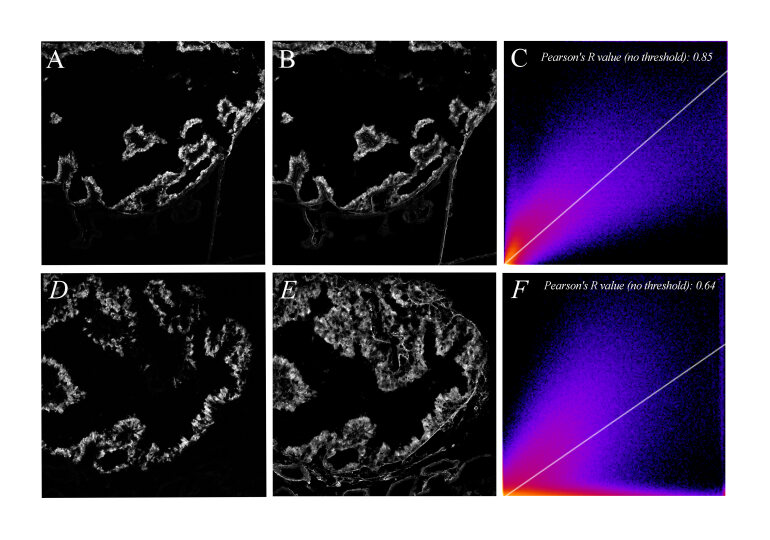

Supplement: FIGURE S1 — Co-expression analysis of exorh-aanat2.1/2 and exorh-rgr in the pineal organ in Atlantic salmon parr. A: Expression of exorh shown in gray scale of confocal image, of double ISH labeling (green channel in Figure 6A1). B: Expression of aanat2.1/2 shown in gray scale (red channel in Figure 6A1). C: 2D pixel intensity histogram, (x-axis is green channel and y-axis is red channel) showing high degree of pixel overlap between the two channels, Pearson’s R value 0,85. D: Expression of exorh shown in gray scale of confocal image, of double ISH labeling (green channel in Figure 5D1). E: Expression of rgr shown in gray scale (red channel in Figure 5D1). F: 2D pixel intensity histogram, (x-axis is rgr and y-axis is exorh) showing some degree of pixel overlap between the two channels, Pearson’s R value 0,64. [file Image1.tif]
