# Supplementary material for: The photoreceptive and neuroendocrine pineal organ of Atlantic salmon
Source: Front Physiol. 2026 May 25;17:1778109. doi: 10.3389/fphys.2026.1778109 (PMC13243063; doi:10.3389/fphys.2026.1778109)
Supplement: Supplementary file 2 [file Table1.docx]

**S1 Table**: Ensembl GeneIDs for the nonvisual opsins and normalized counts.

| **Gene name** | **EnsemblGeneIDv3** | **Counts** |
| --- | --- | --- |
| *exorh* | ENSSSAG00000006623 | 74242 |
| *rgra1* | ENSSSAG00000006385 | 2721 |
| *rgra2* | ENSSSAG00000048215 | 65 |
| *rgrb2* | ENSSSAG00000078574 | 725 |
| *rgrb1* | ENSSSAG00000120288 | 2421 |
| *rrh/peropsin* | ENSSSAG00000005205 | 2260 |
| *tmtopsin1b2* | ENSSSAG00000098456 | 3758 |
| *parapinopsina* | ENSSSAG00000102045 | 1320 |
| *parapinopsinb* | ENSSSAG00000047145 | 571 |
| *parietopsin* | ENSSSAG00000042598 | 1224 |
| Others: |  |  |
| *opn3b* | ENSSSAG00000008619 | 79 |
| *opn7b* | ENSSSAG00000009564 | 58 |
| *opn5b* | ENSSSAG00000044795 | 10 |
| *opn3a* | ENSSSAG00000053835 | 74 |
| *opn4x1b1* | ENSSSAG00000054324 | 29 |
| *tmtopsin1a1* | ENSSSAG00000054346 | 36 |
| *opn7cd2* | ENSSSAG00000055264 | 17 |
| *opn4m1a1* | ENSSSAG00000067894 | 10 |
| *opn6b* | ENSSSAG00000071695 | 108 |
| *opn8a1* | ENSSSAG00000074073 | 17 |
| *opn4x1a* | ENSSSAG00000075477 | 14 |
| *tmtopsin2b* | ENSSSAG00000076875 | 22 |
| *tmtopsin3a2* | ENSSSAG00000108797 | 10 |
| *tmtopsin3a1* | ENSSSAG00000111843 | 10 |
